# Binaural Listening with Head Rotation Helps Persons with Blindness Perceive Narrow Obstacles

**DOI:** 10.3390/ijerph20085573

**Published:** 2023-04-19

**Authors:** Takahiro Miura, Naoyuki Okochi, Junya Suzuki, Tohru Ifukube

**Affiliations:** 1 National Institute of Advanced Industrial Science and Technology (AIST), Kashiwa 277-0882, Japan; 2 Research Center for Advanced Science and Technology (RCAST), The University of Tokyo, Tokyo 153-8904, Japan; 3 Sony Corporation, Tokyo 141-8610, Japan

**Keywords:** visual impairment, obstacle sense, binaural listening, head rotation

## Abstract

Orientation and mobility (O&M) are important abilities that people with visual impairments use in their independent performance of daily activities. In orientation, people with total blindness pinpoint nonsounding objects and sounding objects. The ability to perceive nonsounding objects is called *obstacle sense*, wherein people with blindness recognize the various characteristics of an obstacle using acoustic cues. Although body movement and listening style may enhance the sensing of obstacles, experimental studies on this topic are lacking. Elucidating their contributions to obstacle sense may lead to the further systematization of techniques of O&M training. This study sheds light on the contribution of head rotation and binaural hearing to obstacle sense among people with blindness. We conducted an experiment on the perceived presence and distance of nonsounding obstacles, which varied width and distance, for participants with blindness under the conditions of binaural or monaural hearing, with or without head rotation. The results indicated that head rotation and binaural listening can enhance the localization of nonsounding obstacles. Further, when people with blindness are unable to perform head rotation or use binaural hearing, their judgment can become biased in favor of the presence of an obstacle due to risk avoidance.

## 1. Introduction

To assist people with visual impairments in performing daily activities, various special needs education and rehabilitation methods and assistive devices have been proposed [1,2,3,4,5,6]. Orientation and mobility (O&M) are the primary focus for gait training among people with visual impairments that enable them to acquire independent mobility [7,8]. Orientation refers to the ability to recognize one’s distance and direction relative to observed or remembered objects in one’s surroundings and maintain these spatial relationships as they change during locomotion. Mobility refers to the act of moving through space with precision and efficiency [9,10,11]. When people with visual impairments can systematically integrate these two aspects, their navigation and movement can become purposeful and directional [12]. Practical studies of O&M ability in people with visual impairment have been conducted with children [13,14,15,16,17,18] and with older adults with visual impairment [19,20,21,22]. Additionally, knowledge of training O&M abilities is increasing [23,24,25].

Using orientation, people with total blindness can localize the positions of sounding objects and nonsounding objects [7,8]. For the localization of sounding objects, various studies have reported that the ability to localize sound sources is dependent on the status of the visual impairment. Certain studies have reported that the accuracy of horizontal localization in participants with blindness was the same as or exceeded that of participants with normal vision, whereas the accuracy of participants with low levels of vision was lower [26,27]. Zwiers et al. reported similar findings but found that the level of vertical directional accuracy of localization was lower in people with blindness than in those with normal vision [28].

The ability to perceive nonsounding objects is called *obstacle sense* [29,30,31,32]. Various studies have reported auditory-experienced persons with blindness can recognize the presence of nonsounding obstacles and localize their characteristics using acoustic cues, such as insulation and reflection caused by an obstacle [33,34,35,36,37,38,39,40,41]. Kellogg and Rice investigated the ability of blind persons to discriminate the distance, size, and material of obstacles using obstacle sense and reported that some blind persons can discriminate distance more accurately than those using visual depth perception, and others can distinguish differences between and the hardness of materials [42,43,44]. Strategies for the auditory perception of obstacles include passively listening to environmental sounds to localize them, as well as actively producing cue sounds on their own. The former is often called obstacle sense, while the latter is called echolocation. In the latter case, acoustic factors that enhance the ability to perceive obstacles include footstep sounds [45], the tapping sound of a white cane [46], hand and finger claps [47], and tongue clicks [48,49].

Other factors that may enhance the accuracy of obstacle sense include the individual’s own movement. In particular, behavior that changes the relative distance from the obstacle has been reported to enhance the localization of nonsounding obstacles in the real world. These movements include the transverse and sagittal movements of a person toward obstacles [50], as well as the preceding sound effects when approaching obstacles at a short distance [51]. Meanwhile, even without changing the relative distance, it is possible that head rotation and binaural listening can improve the accuracy of perceiving the distance and direction of obstacles. For source localization, head rotation and binaural listening can improve localization accuracy in terms of direction and distance [52,53,54,55]. Loetscher et al. suggested that head movement arouses attention for spatial perception [56]. With respect to obstacle perception, people with blindness reported that various strategies, such as head rotation and binaural hearing, could improve the subjective presence of obstacles and walls [57]. However, whether head rotation and binaural listening can, in fact, improve the accuracy of obstacle perception remains unclear due to the lack of experimental studies. Clarification of this point may lead to the further systematization of O&M training techniques.

Therefore, this study seeks to elucidate the effects of head rotation and binaural hearing on obstacle sense among people with blindness. In particular, we conducted an experiment on the perceived presence and distance of nonsounding obstacles for participants with blindness under the combined conditions of binaural or monaural hearing and with or without head rotation. In the experiment, the obstacles varied in terms of breadth and distance from the participant.

## 2. Methods

### 2.1. Participants

This study recruited eight individuals with congenital or early blindness and three with acquired blindness aged 22–37 years (average: 30.1 years). All were Japanese persons with total blindness (female: 3; male: 8). The participants had normal hearing and within normal limits (<20 dB in hearing level) of pure-tone audiometry (Rion AA-75).

### 2.2. Experimental Setup

**Figure 1:**
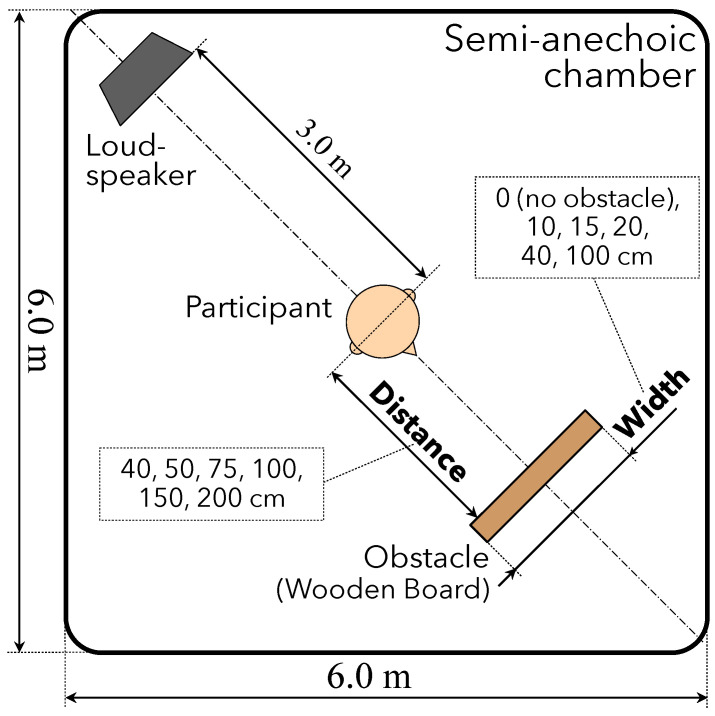
Experimental environment for perception of the presence and distance of obstacles.

Figure 1 presents the experimental environment. A loudspeaker (Tannoy System 800) was placed 3.0 m from the center of a semianechoic chamber (6.0 m × 6.0 m × 2.5 m; reverberation time: 1.8 s; background noise level: 40–45 dB). We employed pink noise with a frequency range of 20–20,000 Hz and played it from the loudspeaker because ambient noise in the real environment is closer to pink noise than it is to white noise. Some experimental studies on sound localization have employed pink noise as a sound source [54,58,59,60]. The frequency response of a loudspeaker is generally flat, according to the manufacturer’s specification. At the opposite side of the loudspeaker, we placed an obstacle (a wooden board) with a height and depth of 67 and 1.0 cm, respectively. The obstacle varied at distances of 40, 50, 75, 100, 150, and 200 cm and at widths of 10, 15, 20, 40, and 100 cm. The positions of the loudspeaker and an obstacle were determined based on similar experiments reported previously [32,34,51].

We employed this range of distances because the area approximately 3.0 m from the obstacle is defined as the position of the *first perception*, at which a person with blindness can recognize the presence of an obstacle. Meanwhile, the area 0.3 m from an obstacle is defined as the *final appraisal*, wherein the person with blindness deems they can no longer approach [2,32,51]. The reason for adopting this range of widths was based on preliminary testing of a participant with blindness that was based on a study by Nakamura-Funaba et al. [61]. The authors reported that people with blindness experience difficulty in recognizing narrow objects, such as utility poles or antenna supports.

### 2.3. Procedure

Before the experiment, the participants were seated at the center of the chamber with a loudspeaker located behind them for each trial and were seated facing the obstacle. The sound intensity of the loudspeaker was set at a comfortable level (Range: 55–65 dB).

In the experimental session, the participants were first requested to wear earmuffs to avoid hearing distracting sounds from the environment. Simultaneously, the experimenter randomly selected an obstacle with various widths and placed it at one of the distances previously discussed. Moreover, the center of the obstacle height was placed at the ear level of the participant. Finally, the experimenter frequently moved or removed the obstacle at random times to control for false-positive reactions.

Next, the loudspeaker was used to generate pink noise. When the experimenter gently tapped the shoulder of the participants, they removed the earmuffs and listened carefully to the environmental sound under one of the following conditions: binaural/monaural listening with/without head rotation. Under monaural conditions, the participants selected which ear to insert an earplug before the experiment.

Afterward, participants provided the localization certainty and localized distance of the obstacle. The localization certainty denotes how strongly the participant perceived the presence of the obstacle, rated using a 5-point Likert-type scale indicated using their fingers. A rating of 5 indicates that they felt nearly 80%–100% of the presence of the obstacle in front of them. However, where participants did not perceive an obstacle responded with 0. They also orally provided localized distance (in cm).

The experiment was composed of the preferred number of practices and 124 sets (= (5 (obstacle width) × 6 (obstacle distance) + 1 (no obstacle)) × 4 (listening condition)) for the actual tests for each participant.

### 2.4. Analysis

Localization certainty and localized distance were first plotted in the presence and absence of obstacles.

Based on the localization certainty, a confusion matrix (two rows by two columns) of perceived obstacle presence/absence and experimental conditions with/without a real obstacle was created after defining the response of obstacle presence/absence as to whether the localization certainty of the obstacle was 0 or 1–5. The discrimination power $d^{\prime }$ and response bias β were then calculated based on signal detection theory [62]. $d^{\prime }$ decreases and increases in the number of cases where localization certainty is 0 and an obstacle is present or conversely when the localization certainty is 1–5 where no obstacle was present. A value of β greater or smaller than 1.0 indicates that the response is biased toward one side or the other. An analysis of variance (ANOVA) was employed to identify significant differences among responses to $d^{\prime }$ and β and to examine the main effects of head rotation and binaural listening for these responses. Before the ANOVA was performed, the aligned rank transform (ART) [63,64] was conducted on the scales, as the responses were non-normally distributed. Then, the significance of the main effects was determined using post hoc multiple comparison methods based on the least-square means and Tukey’s multiplicity adjustment [65,66].

Regarding the raw responses of the localization certainty, multiple ordinal logistic regression (OLR) was used to examine the significant contribution ratio of the experimental conditions, such as obstacle width and distance, head rotation, and binaural listening. Then, the log odds ratio for each attribute was calculated to determine the degree of relevance of the responses. After this, the effect of the factors was discussed based on the presence or absence of significant differences and magnitude of the log odds ratio. The effect of one factor is greater than that of other factors when the log odds ratio of that factor was greater than 0, and vice versa.

To clarify the significant main effects and interactions for localized distance and the ratio of localized to actual distances, along with the statistical analyses methods described above, we employed ANOVA with ART [63,64]. The significance of the main effects was also determined using Lenth’s method [65,66].

## 3. Results and Discussion

### 3.1. Discrimination Power and Detection Bias in Identifying the Obstacle

**Figure 2:**
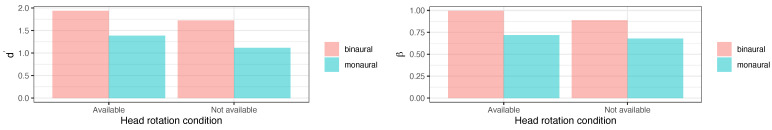
Discrimination power $d^{\prime }$ (**left**) and detection bias β (**right**) with and without head rotation in binaural and monaural hearing.

Figure 2 presents the discrimination power $d^{\prime }$ and response bias β calculated on the basis of signal detection theory in the presence and absence of obstacles.

The results demonstrated that the value of $d^{\prime }$ tended to increase with head rotation instead of without head rotation and with binaural hearing instead of unilateral hearing. Additionally, when participants used binaural hearing with head movement, β≃1 was obtained; thus, the response bias between the subjective presence and absence of the obstacle was nearly eliminated. Alternatively, the value of β was generally smaller than 1.0 in the case of unilateral hearing or no head movement. This finding indicates a bias in determining the presence of the obstacle. Thus, in general, head rotation and binaural hearing could be useful factors for the accurate perception of the presence of obstacles. By contrast, where these conditions are difficult to perform, bias may exist for determining the presence of obstacles from various perspectives, such as risk avoidance.

An ANOVA performed on changes in $d^{\prime }$ and β confirmed a significant main effect for all factors, including obstacle width, obstacle distance, availability of head movement, and binaural/monaural hearing (p<0.05). The statistical value of these significant factors in $d^{\prime }$ was as follows: obstacle width: $F\left(1,4\right)=10.14,p<0.001,\mathrm{partial}\;\eta^{2}=0.40$, obstacle distance: $F\left(1,5\right)=7.26,p<0.001,\mathrm{partial}\;\eta^{2}=0.38$, availability of head movement: $F\left(1,1\right)=6.49,p=0.013<0.05,\mathrm{partial}\;\eta^{2}=0.098$, binaural/monaural hearing: $F\left(1,1\right)=28.68,p=0.008<0.01,\mathrm{partial}\;\eta^{2}=0.42$. Also, those in β were as follows: obstacle width: $F\left(1,4\right)=4.90,p=0.002<0.01,\mathrm{partial}\;\eta^{2}=0.24$, obstacle distance: $F(1,5)=4.95,p<0.001,\mathrm{partial}\;\eta^{2}=0.29$, availability of head movement: $F(1,1)=6.72,p=0.012<0.05,\mathrm{partial}\;\eta^{2}=0.10$, binaural/monaural hearing: $F(1,1)=63.08,p=0.001,\mathrm{partial}\;\eta^{2}=0.51$.

The interaction between obstacle width and distance was significant for $d^{\prime }$ ($F(1,20)=2.25,p=0.008<0.01,\mathrm{partial}\;\eta^{2}=0.43$) and had a significant trend for β ($F\left(1,20\right)=1.68,p=0.06<0.10,\mathrm{partial}\;\eta^{2}=0.36$). The results of multiple comparisons illustrated that the value of $d^{\prime }$ was significantly larger under the condition with head movement than that without head movement (t(60)=2.55,p=0.013<0.05,d=0.47), whereas the value of β was significantly larger and closer to 1.0 (t(60)=2.59,p=0.012<0.05,d=0.47). Additionally, the $d^{\prime }$ value was significantly larger under the condition with binaural hearing than that with unilateral hearing (t(60)=5.35,p<0.001,d=0.98), whereas the β value was also significantly larger and closer to 1.0 (t(60)=7.94,p<0.001,d=1.45).

**Figure 3:**
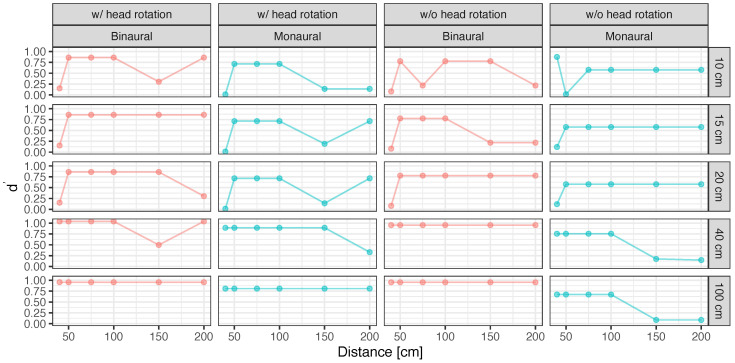
Discrimination power $d^{\prime }$ with and without head rotation with binaural and monaural hearing for various obstacle distances and widths.

**Figure 4:**
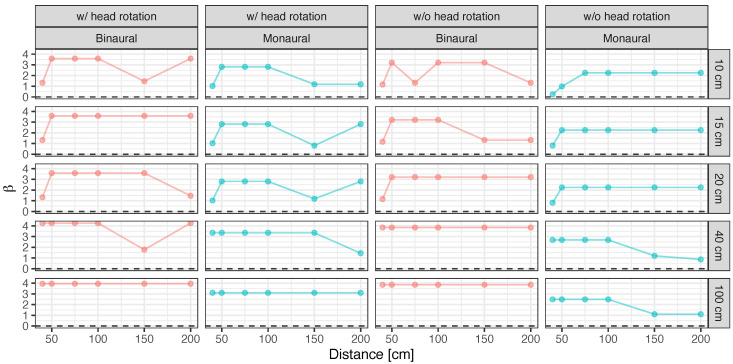
Detection bias β with and without head rotation in binaural and monaural hearing for various obstacle distance and width.

Figure 3 and Figure 4 demonstrate $d^{\prime }$ and β for the various distances and widths of the obstacle with and without head movement and with binaural and unilateral hearing, respectively. As shown in Table 1, the results of multiple comparisons indicate that $d^{\prime }$ and β are generally significantly larger and smaller, respectively (p<0.01), for widths of 40 and 100 cm than for widths of 10, 15, and 20 cm.

For distance, $d^{\prime }$ was significantly larger, whereas bias β was significantly smaller at 50, 75, and 100 cm than at 40 cm (p<0.05 for all distances), as shown in Table 2. Moreover, no significant differences were observed in $d^{\prime }$ and bias between distances of 150 and 200 cm (p>0.10 for all distances) at an obstacle distance of 40 cm. Notably, a significant interaction was confirmed for obstacle width and distance that was only seen when the distance of the obstacle was 40 cm. Table 3 shows the effect sizes of obstacle width conditions when the obstacle distance were 40 cm. In this condition, similar to the results in Table 4, differences with significance or marginal significance of $d^{\prime }$ and β were confirmed among obstacle widths of 10, 15, and 20 cm and 40 and 100 cm (p<0.05 or p<0.10). The effect size in Table 3 is larger than the effect size in cases where the differences are significant, in Table 3 and Table 4. Thus, the closer the distance between the person and the obstacle, the greater the variation in obstacle detection and presence bias due to the differences in obstacle width.

At distances of 40, 150, and 200 cm, the participants could not accurately localize the presence of the obstacle and tended to display a bias toward the presence of the obstacle. This suggests that perceiving the presence of obstacles may be difficult, not only when objects are far away but also when they are extremely close. As the distance between the obstacle and the participant becomes closer, the echo time is also shortened because the acoustic path between the direct sound coming from behind the participant and the reflected sound from the obstacle in front of them becomes shorter. This shortening of the time interval increases the interval between the dip frequencies in acoustic coloration (the frequency response of a comb filter caused by interference between direct and reflected sounds). Miura et al. found that the discrimination threshold of acoustic coloration, or timbral changes caused by the interference of background sound and reflected sounds from an obstacle and varied with the distance of the obstacle, becomes larger as the distance increases to greater than almost ∼600 Hz, which corresponds to the *final appraisal* (an approximately 30 cm distance between the participant and the obstacle) [34]. It has also been reported that people with visual impairments perceive difficulty in approaching an obstacle at an approximate distance of 30 cm, which is the *final appraisal* distance [32,51], so it is possible that in such a case, the participants would not mainly use auditory information but would stretch their arms out to touch the obstacle to judge its presence.

Another possible explanation is that where the width of the obstacle is small and the distance is close, the head interfered with the source of the sound behind the participant in the experimental setup, which reduced the gain of reflected sound. The significant interaction between obstacle width and distance was confirmed, indicating that it is difficult to perceive the presence of a narrow obstacle when the obstacle is close. However, based on the results shown in Figure 2, head movement and binaural hearing may facilitate perception to a certain extent even in such cases.

### 3.2. Localization Certainty

**Figure 5:**
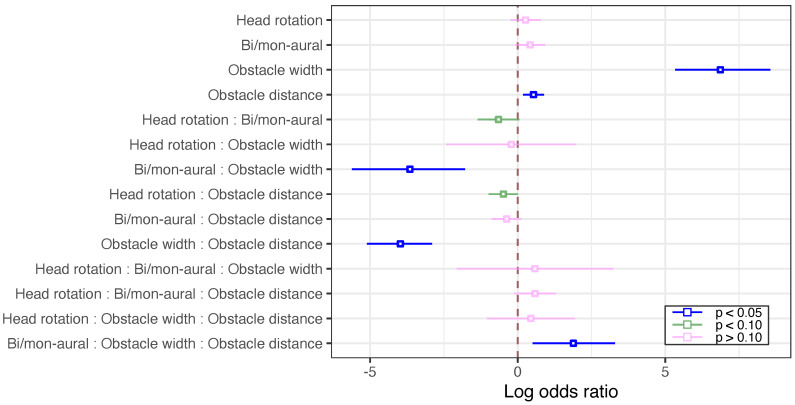
Log odds ratios of the main effects and interactions to localization certainty on the following factors: availability of head rotation, binaural/monaural hearing, obstacle width, and obstacle distance.

Figure 5 show the log odds ratio for the experimental conditions. The OLR results of the responses indicated significant main effects for obstacle width and distance (p<0.05) but nonsignificant for availability of head rotation and binaural/monaural hearing (p>0.10). However, significant or marginally significant interactions regarding the availability of head rotation and binaural/monaural hearing were observed with those of availability of head rotation and binaural/monaural hearing (p<0.10), binaural/monaural hearing and obstacle width (p<0.05), availability of head rotation and obstacle distance (p<0.10), and binaural/monaural hearing and obstacle width and distance (p<0.05). Thus, all of the experimental variables we employed could have contributed to the sense of obstacle presence. In the cases where a significant main effect was observed, positive log odds ratios were obtained for obstacle width and obstacle distance, while negative log odds ratios were observed for interactions of two factors with significance or marginal significance. For the absolute value of the log odds ratios, obstacle width was the largest among the attributes with positive values. However, for negative values, the interaction of binaural/monaural hearing and obstacle width was large, whereas the interaction of presence of head movement and obstacle distance was small.

**Figure 6:**
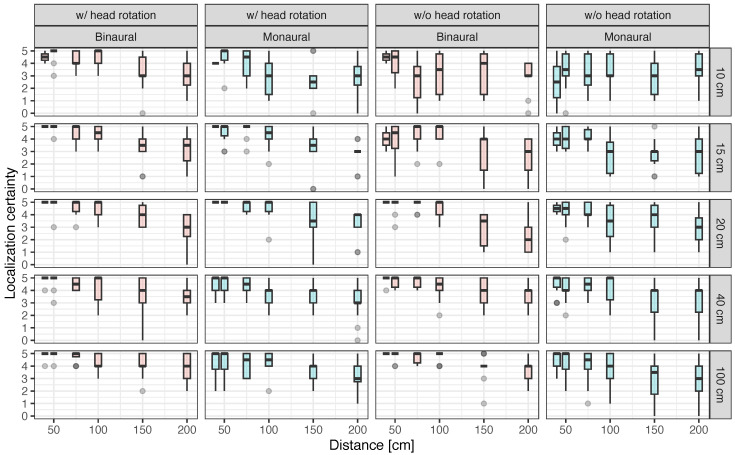
Localization certainty as a function of distance between the participant and obstacle under the conditions with and without head rotation in binaural and monaural hearing.

Figure 6 illustrates the localization certainty in the presence of obstacles. In general, localization certainty tended to increase with decreases in the distance between obstacles and the increases in the width of the obstacles. Further, the presence of obstacles was evaluated as low and varied under the conditions without head rotation and the monaural conditions, particularly when the obstacle width and distance were 10 cm and under 100 cm, respectively. However, variation in judgment increased with the addition of the condition of monaural hearing.

According to the localization certainty represented in Figure 6, the variation in localization certainty under the condition without head rotation was larger when the obstacle width was less than 15–20 cm. The average Japanese person’s head width (bitragion breadth) is 14.86 ± 1 cm [68]. When the obstacle width is smaller than this head width, the intensity of the reflected sound from the obstacle may drastically decrease due to head shadow [69]. Thus, the presence of the obstacle at obstacle widths of less than 20 cm is not varied because the change in sound intensity between the far and near distances from the obstacle is nearly the same. However, note that the task in our experiment was to localize obstacles by passively listening with pink noise. In the case of active sound localization, the characteristics of the auditorily perceivable obstacle may differ depending on the position and type of the generated sound, such as footsteps, the tapping of a white cane, hand claps, finger snaps, and tongue clicks.

For head movement, the change in localization certainty with respect to distance was upward-sloping regardless of the width of the obstacle, as shown in Figure 6. By rotating the head, the position of the ears changes relative to the reflective surface of the obstacle. Consequently, the power of the reflected sound lost during diffraction is reduced relative to the case where both ear axes of the head are parallel to the obstacle. Thus, changes of interaural differences due to head movement may contribute to the increase in the subjective presence of the obstacle. Indeed, some participants noted that they routinely performed actions such as moving their head to listen carefully to the sounds around them and recognize spatial information.

#### 3.2.1. Localized Distance

ANOVA on localized distance confirmed significant or marginally significant main effects for obstacle width ($F\left(1,4\right)=39.19,p<0.001,\mathrm{partial}\;\eta^{2}=0.12$), obstacle distance ($F\left(1,5\right)=158.8,p<0.001,\mathrm{partial}\;\eta^{2}=0.41$), and availability of head movement ($F(1,1)=3.60,p=0.058<0.10,\mathrm{partial}\;\eta^{2}=0.003$). However, there were no significant main effect for binaural/monaural hearing (F(1,1)=2.166,p=0.1414>0.10,partial$\eta^{2}=0.002$). We confirmed significant interactions for the availability of head movement and obstacle width ($F\left(1,4\right)=4.28,p=0.002<0.01,\mathrm{partial}\;\eta^{2}=0.015$), binaural/monaural hearing and obstacle distance ($F\left(1,5\right)=2.99,p=0.011<0.05,\mathrm{partial}\;\eta^{2}=0.013$), and obstacle width and distance ($F\left(1,20\right)=2.91,p<0.001,\mathrm{partial}\;\eta^{2}=0.049$). Therefore, all of the experimental variables we employed could contribute to the localized distance. However, because the effect size partial $\eta^{2}$ was too large to moderate for obstacle distance and width but too small to negligible for availability of head movement and significant interactions related to the head movement and binaural/monaural hearing, head movement and binaural/monaural hearing seemed to have a partial influence on distance determination.

**Figure 7:**
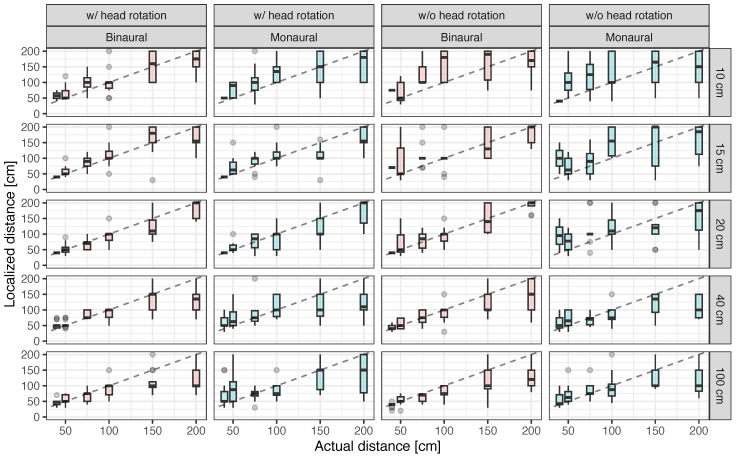
Localized distance as a function of distance between participant and obstacle under conditions with and without head rotation in binaural and monaural hearing. The dashed lines represent cases where the actual and localized distances are the same.

Figure 7 presents the localized distance provided by the participants. In general, the localized distance increased with the actual distance between the participant and an obstacle. Additionally, the variation in localized distance seemed to be larger when the participants did not conduct head movement and/or when they listened monaurally, especially when the actual distance to the obstacle was less than 50 cm. When the obstacle width was greater than the head width (40 cm and 100 cm), the localized distance could be smaller than the actual distance when the obstacle distance was greater than 100 cm, regardless of the availability of head movement and binaural/monaural hearing.

#### 3.2.2. Ratio of Localized to Actual Distances

ANOVA on the ratio of localized to actual distances confirmed significant or marginally significant main effects for binaural/monaural hearing ($F\left(1,1\right)=15.7,p<0.001,\mathrm{partial}\;\eta^{2}=0.014$), obstacle width ($F\left(1,4\right)=13.85,p<0.001,\mathrm{partial}\;\eta^{2}=0.047$), and obstacle distance ($F\left(1,5\right)=70.04,p<0.001,\mathrm{partial}\;\eta^{2}=0.24$). However, there were no significant main effects for the availability of head movement ($F\left(1,1\right)=2.69,p=0.101>0.10,\mathrm{partial}\;\eta^{2}=0.0024$). Meanwhile, we confirmed significant interactions for the availability of head movement and obstacle width ($F\left(1,4\right)=7.08,p<0.001,\mathrm{partial}\;\eta^{2}=0.0247$), binaural/monaural hearing and obstacle distance ($F\left(1,5\right)=7.51,p<0.001,\mathrm{partial}\;\eta^{2}=0.032$), and obstacle width and distance ($F\left(1,20\right)=1.65,p=0.036<0.05,\mathrm{partial}\;\eta^{2}=0.029$). Therefore, similar to the results for localized distance, all of the experimental variables we employed could contribute to accurate distance localization. However, significant main effects for binaural/monaural listening were confirmed in the ratio of localized to actual distances but not in the localized distance condition. Conversely, a main effect with marginal significance of the availability of head movement was found in the localized distance condition but not in the ratio condition. Thus, while binaural listening may be important for accurate distance localization, head movement can increase the consistency of distance judgments.

**Figure 8:**
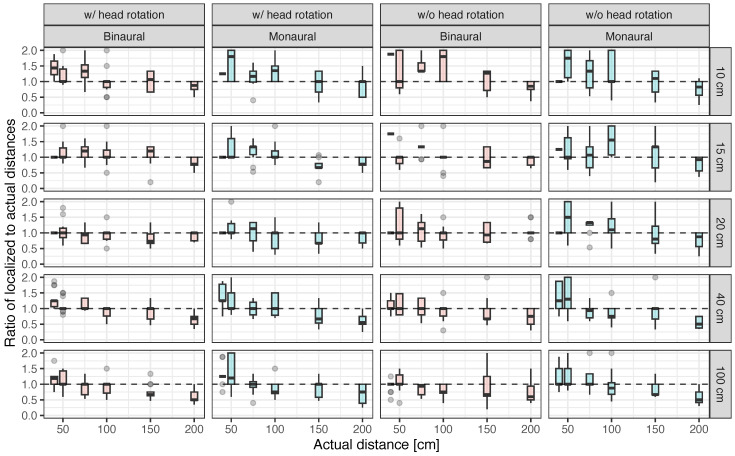
Ratio of localized to actual distances as a function of distance between the participant and obstacle under conditions with and without head rotation in binaural and monaural hearing.

Figure 8 shows the ratio of localized to actual distances in various conditions of obstacle width, availability of head movement, and binaural/monaural listening. Overall, localized distance tended to be rated as further than the actual distance when the obstacle distance was less than 100 cm, whereas it tended to be rated as closer than the actual distance when the obstacle distance was more than 100 cm. It was also found that the variation in the ratio of localized and actual distances was greater when the participants listened monaurally and without head movement. However, when the obstacle width was 15 or 20 cm, the ratio of localized to actual distances was close to 1.0, regardless of the obstacle’s distance, when the participants were allowed to move their heads and listen binaurally. Therefore, the distance for obstacles whose widths approximately equal the width of the head may be the easiest to perceive. The reason for this is a topic for further study.

## 4. Limitations

In this study, we asked blind persons to localize obstacles that did not emit sound in an environment with background pink noise. Although we focused on the case where blind people moved their heads and listened binaurally/monaurally, some studies have found that blind people sometimes actively emit sounds to attempt echolocation in their daily environments. The sounds used in these situations are footstep sounds [45], the tapping sound of a white cane [46], hand claps and finger snaps [47], and tongue clicks [48,49], as mentioned above. These sounds are not emitted continuously but often intermittently or once. The results obtained using these sounds may differ from those reported in this paper. Moreover, although our study examined obstacle perception using head movement, we did not examine the sounds and detailed perceptual phenomena heard during such movements. The above is worth further investigation to determine how the passive and active obstacle perception are combined.

## 5. Conclusions

To elucidate the effects of head movement and binaural hearing on obstacle sense among persons with blindness, this study conducted an experiment to determine the localization of nonsounding obstacles of varied distances and widths. The results demonstrated that head rotation and binaural hearing can enhance localization of nonsounding obstacles and contribute to highly accurate distance localizations of obstacles. When people with blindness cannot perform head rotation or binaural hearing, bias was observed in their judgment in favor of the presence of the obstacle from the viewpoint of risk avoidance. The present study was conducted in a context in which the participants did not approach the obstacle. Thus, the examination of strategies for enhancing the presence of obstacles during walking remain as an interesting avenue for future studies.

## Figures and Tables

**Table 1:** Cohen’s *d* (|d|) of $d^{\prime }$ (**left**) and β (**right**) among the obstacle width (in cm) conditions. We used the criterion of Cohen’s effect size indices [67].

$d^{\prime }$						*β*				
	10	15	20	40			10	15	20	40
**15**	0.221					**15**	0.225			
**20**	0.405	0.184				**20**	0.312	0.087		
**40**	1.47 ***	1.25 ***	1.07 **			**40**	1.48 ***	1.25 ***	1.17 **	
**100**	1.43 ***	1.21 ***	1.02 **	0.047		**100**	1.49 ***	1.27 ***	1.18 **	0.014

Significance: ***: p<0.001, **: p<0.01, *: p<0.05, †: p<0.10. Cohen’s *d*: Large: |d|≥0.80, Medium: |d|≥0.50, Small: |d|≥0.20.

**Table 2:** Cohen’s *d* (|d|) of $d^{\prime }$ (**left**) and β (**right**) among the obstacle distance (in cm) conditions. We used the criterion of Cohen’s effect size indices [67].

$d^{\prime }$							*β*					
	40	50	75	100	150			40	50	75	100	150
**50**	0.543 **						**50**	0.724 ***				
**75**	0.275 ***	0.912					**75**	0.314 **	0.909			
**100**	0.661 ***	−0.024	50				**100**	0.624 ***	−0.029	50		
**150**	0.637	0	0	0.851 *			**150**	0.595	0	0	0.992 *	
**200**	0.936	0	1	0.479 ^†^	0.526		**200**	0.938	0	0.999	0.18 ^†^	0.215

Significance: ***: p<0.001, **: p<0.01, *: p<0.05, †: p<0.10. Cohen’s *d*: Large: |d|≥0.80, Medium: |d|≥0.50, Small: |d|≥0.20.

**Table 3:** Cohen’s *d* (|d|) of $d^{\prime }$ (**left**) and β (**right**) among the obstacle width (in cm) conditions when obstacle distance was 40 cm. We used the criterion of Cohen’s effect size indices [67].

$d^{\prime }$						*β*				
	10	15	20	40			10	15	20	40
**15**	0.225					**15**	0.141			
**20**	0.355	0.13				**20**	0.25	0.109		
**40**	2.35 **	2.58 **	2.71 **			**40**	2.35 *	2.58 *	2.71 *	
**100**	2.21 *	2.44 *	2.57 *	0.137		**100**	2.21 ^†^	2.24 ^†^	2.57 ^†^	0.154

Significance: **: p<0.01, *: p<0.05, †: p<0.10. Cohen’s *d*: Large: |d|≥0.80, Medium: |d|≥0.50, Small: |d|≥0.20.

**Table 4:** Cohen’s *d* (|d|) of localized distance among the obstacle width (**left**) and the obstacle distance (**right**) conditions. We used the criterion for Cohen’s effect size indices [67].

Obstacle Width						Obstacle Distance					
	10	15	20	40			40	55	75	100	150
**15**	0.163					**50**	0.461 *				
**20**	0.371 *	0.208				**75**	1.08 ***	0.619 ***			
**40**	2.35 ***	2.58 ***	2.71 ***			**100**	1.63 ***	1.17 ***	0.548 ***		
**100**	2.21 ***	2.44 ***	2.57 ***	0.029		**150**	2.25 ***	1.79 ***	1.17 ***	0.627 ***	
						**200**	2.62 ***	2.16 ***	1.54 ***	0.993 ***	0.366 **

Significance: ***: p<0.001, **: p<0.01, *: p<0.05, †: p<0.10. Cohen’s *d*: Large: |d|≥0.80, Medium: |d|≥0.50, Small: |d|≥0.20.

## Data Availability

Not applicable.
